# Identification and functional analysis of missense mutations in the lecithin cholesterol acyltransferase gene in a Chilean patient with hypoalphalipoproteinemia

**DOI:** 10.1186/s12944-019-1045-0

**Published:** 2019-06-05

**Authors:** Hugo E. Tobar, Luis R. Cataldo, Trinidad González, Ricardo Rodríguez, Valentina Serrano, Antonio Arteaga, Ana Álvarez-Mercado, Carlos F. Lagos, Lucas Vicuña, José P. Miranda, Ana Pereira, Carolina Bravo, Concepción M. Aguilera, Susana Eyheramendy, Ricardo Uauy, Álvaro Martínez, Ángel Gil, Omar Francone, Attilio Rigotti, José L. Santos

**Affiliations:** 1 Department of Nutrition, Diabetes and Metabolism, School of Medicine, Pontificia Universidad Católica de Chile, Santiago, Chile; 2 Pfizer Global Research and Development, San Diego, USA; 3 INYTA, University of Granada, Granada, Spain; 4 Department of Biochemistry and Molecular Biology II, Institute of Nutrition and Food Technology “José Mataix”, Center of Biomedical Research, University of Granada, Granada, Spain; 5 INTA, Universidad de Chile, Santiago, Chile; 6 División de Pediatría, Escuela de Medicina, Pontificia Universidad Católica de Chile, Santiago, Chile; 7 Departamento de Estadísticas, Facultad de Matemáticas, Pontificia Universidad Católica de Chile, Santiago, Chile; 8 Facultad de Medicina y Ciencia, Universidad San Sebastián, Campus Los Leones, Santiago, Chile; 9 Centro de Nutrición Molecular y Enfermedades Crónicas, Escuela de Medicina, Pontificia Universidad Católica de Chile, Santiago, Chile; 10 Fundación Ciencia & Vida, Santiago, Chile

**Keywords:** Lecithin-cholesterol acyltransferase, HDL-cholesterol, Variants, Hypoalphalipoproteinemia

## Abstract

**Background:**

Lecithin-cholesterol acyltransferase (LCAT) is a plasma enzyme that esterifies cholesterol in high- and low-density lipoproteins (HDL and LDL). Mutations in LCAT gene causes familial LCAT deficiency, which is characterized by very low plasma HDL-cholesterol levels (Hypoalphalipoproteinemia), corneal opacity and anemia, among other lipid-related traits. Our aim is to evaluate clinical/biochemical features of a Chilean family with a proband showing clinical signs of familial LCAT deficiency, as well as to identify and assess the functional effects of LCAT mutations.

**Methods:**

An adult female proband with hypoalphalipoproteinemia, corneal opacity and mild anemia, as well as her first-degree relatives, were recruited for clinical, biochemical, genetic, *in-silico* and in-vitro LCAT analysis. Sequencing of exons and intron-exon boundaries was performed to identify mutations. Site-directed mutagenesis was carried out to generate plasmids containing cDNA with wild type or mutant sequences. Such expression vectors were transfected to HEK-239 T cells to asses the effect of LCAT variants in expression, synthesis, secretion and enzyme activity. *In-silico* prediction analysis and molecular modeling was also used to evaluate the effect of LCAT variants.

**Results:**

LCAT sequencing identified rare p.V333 M and p.M404 V missense mutations in compound heterozygous state in the proband, as well the common synonymous p.L363 L variant. LCAT protein was detected in proband’s plasma, but with undetectable enzyme activity compared to control relatives. HEK-293 T transfected cells with vector expression plasmids containing either p.M404 V or p.V333 M cDNA showed detectable LCAT protein expression both in supernatants and lysates from cultured cells, but with much lower enzyme activity compared to cells transfected with the wild-type sequence. Bioinformatic analyses also supported a causal role of such rare variations in LCAT lack of function. Additionally, the proband carried the minor allele of the synonymous p.L363 L variant. However, this variant is unlikely to affect the clinical phenotype of the proband given its relatively high frequency in the Chilean population (4%) and its small putative effect on plasma HDL-cholesterol levels.

**Conclusion:**

Genetic, biochemical, in vitro and in silico analyses indicate that the rare mutations p.M404 V and p.V333 M in LCAT gene lead to suppression of LCAT enzyme activity and cause clinical features of familial LCAT deficiency.

**Electronic supplementary material:**

The online version of this article (10.1186/s12944-019-1045-0) contains supplementary material, which is available to authorized users.

## Background

Blood cholesterol transport is performed, mainly by two lipoproteins: Low Density Lipoproteins (LDL) that participated in cholesterol flux from the liver to peripheral tissues and High-Density Lipoproteins (HDL) that mediate mobilization from peripheral tissues to the liver for excretion into bile and to steroidogenic tissues for steroid hormone synthesis [1, 2]. Lecithin-cholesterol acyltransferase (LCAT) is a plasma soluble enzyme that esterifies free cholesterol incorporated into HDL surface, generating cholesteryl esters that enter into hydrophobic HDL allowing its maturation to spherical particles [3, 4]. Mutations in the LCAT gene (gene ID = 3931) have been related to the severe LCAT deficiency, known as Familial LCAT deficiency (FLD) (OMIM#245900) and to the partial LCAT deficiency, referred to as Fish eye disease (OMIM#136120). Both diseases are characterized by very low or undetectable HDL-cholesterol levels, also called hypoalphalipoproteinemia, as well as corneal opacity and other lipid-related traits, whereas FLD patients also exhibit hemolytic anemia, proteinuria, and renal dysfunction [5, 6].

The aim of this study was to identify causing-disease mutations in the LCAT gene and characterize their functional effects on LCAT activity in a Chilean patient with LCAT deficiency phenotype (hypoalphalipoproteinemia, corneal opacity, multiple lipid abnormalities, mild anemia and without proteinuria) together with her first-degree relatives. Herein, we identified two rare genetic variants p.V333 M and p.M404 V in a Chilean patient with symptoms of FLD. In this research, we demonstrated that the undetectable plasma HDL-cholesterol levels found in this patient are determined by p.M404 V and p.V333 M LCAT gene mutations that abolishes cholesterol esterification activity, without affecting protein expression, synthesis and secretion. In addition, the middle-low frequency variant p.L363 L (rs5923) was also found in this family. However, it is unlikely that this variant affects the severe phenotype of the proband given its relatively high frequency in the Chilean population and its small putative effect on plasma HDL-cholesterol levels, even considering the nominal significant genotype-phenotype association found in participants from the population-based “Growth and Obesity Chilean Cohort Study” [7].

## Subjects and methods

### Family under study and biochemical measurements

**Fig. 1:**
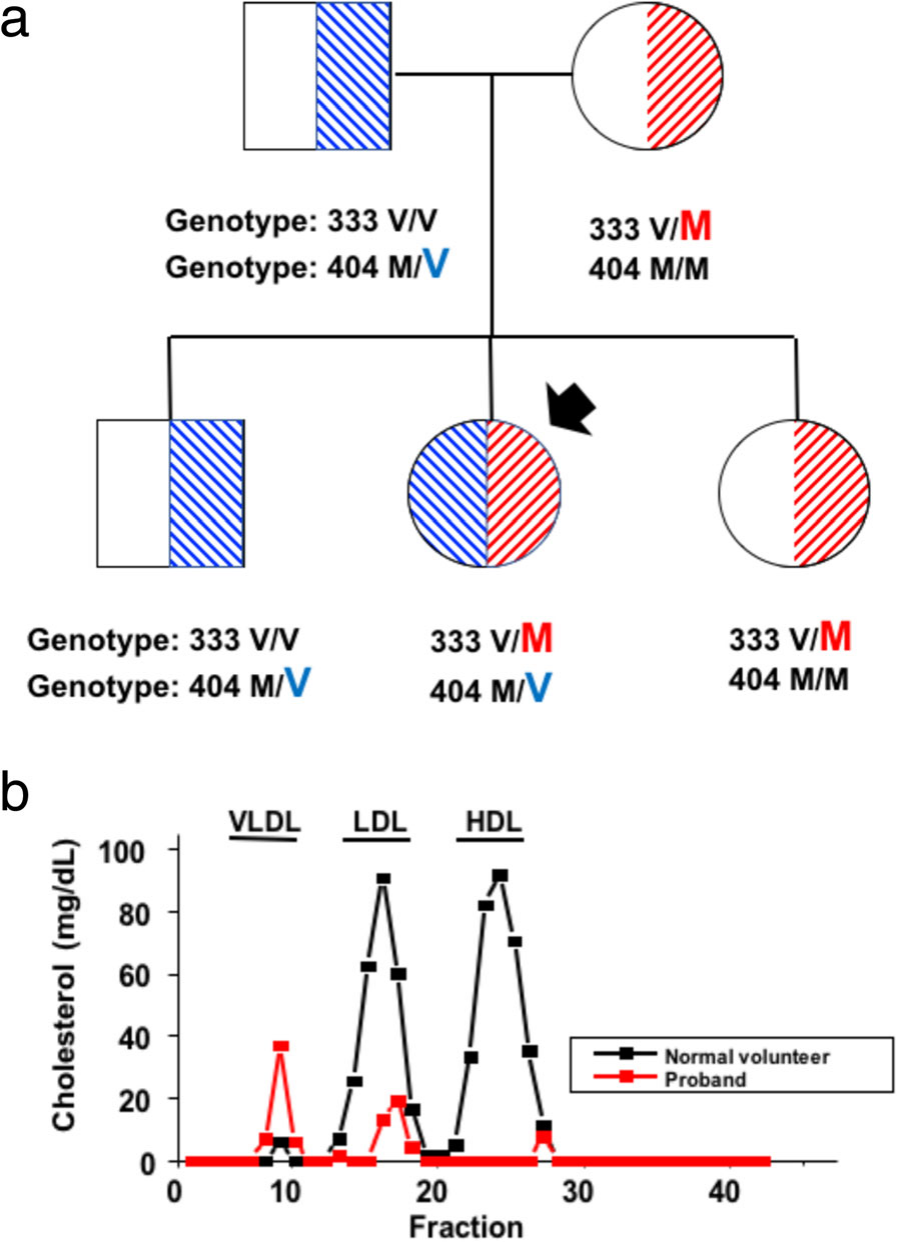
LCAT mutations found in a Chilean patient with hypoalphalipoproteinemia and her relatives. (a) Each family member is shown with his/her genotype in for p.V333 M and p.M404 V mutations. Proband is indicated by a black arrow. (b) The chart shows FPLC fractions for HDL, LDL and VLDL lipoproteins in the proband, indicating near-absence of HDL-lipoproteins and very low levels of LDL-lipoproteins

A 36 years-old female patient with hypoalphalipoproteinemia (HDL-cholesterol = 3 mg/dL) and her first-degree relatives were ascertained from the clinical setting of the School of Medicine of Pontificia Universidad Católica de Chile in the year 2009. Clinical examination revealed clear features of familial LCAT deficiency in the proband, with pronounced corneal opacity, mild anemia and without proteinuria. Standard plasma lipid profile was measured in the laboratory of the School of Medicine at the Pontificia Universidad Católica de Chile (http://redsalud.uc.cl/ucchristus/laboratorio-clinico/). Besides undetectable plasma HDL-cholesterol levels, blood lipid profile was characterized by LDL-cholesterol of 17 mg/dL and plasma triglycerides of 387 mg/dL. Her father (78 years-old at the time of the study), mother (75 years-old), sister (51 years-old) and brother (50 years-old) were apparently healthy, with normal plasma lipid profile and without any clinical features of total or partial LCAT deficiency. Additionally, cholesterol ester/free cholesterol levels were measured by Fast-Protein Liquid Cromatography (FPLC) as an indirect measure of the activity of LCAT on the different lipoprotein fractions. Compared to controls, very low values of esterified cholesterol in HDL, VLDL and LDL fractions were found. The high levels of plasma VLDL-cholesterol fund in the FPLC of the proband are concordant with her high plasma triglyceride levels (387 mg/dL) (Fig. 1b and Additional file 7: Table S1).

### DNA sequencing of the LCAT gene

Genomic DNA was extracted from blood samples with QIAamp Blood Mini Kit (Qiagen; Hilden, Germany). Amplification of six exons and intron-exon boundaries of LCAT was performed using primers previously described by Calabresi et al. [8]. For all amplicons, bidirectional Sanger sequencing was performed by Macrogen (Seoul, Rep. of Korea), and compared to the LCAT consensus sequence deposited in NCBI database (https://www.ncbi.nlm.nih.gov/). Specific amplification of LCAT exon 6 was performed to develop a method for the rapid detection of c.997G > A (p.V333 M) or c.1210A > G (p.M404 V) mutations, that were found in the proband (see “Results” section). The primer sequences used were: forward (5′-TGCAGACCTGCACTTTGAGG-3′) and reverse (5′-TAGTGCCTCCCTTCAACCTGA-3′). For Restriction Fragment Length Polymorphism (RFLP) analysis, we used DraIII-HF restriction enzyme (New England Biolabs, Massachusetts, USA) in order to identify c.997G > A variant (p.V333 M) and HpyCH4IV restriction enzyme (New England Biolabs, Massachusetts, USA) for detection of the c.1210A > G variant (p.M404 V). Visualization of restriction fragments was carried out via DNA electrophoresis on 3% agarose gels (SeaKen, Lonza, Rockland, ME, USA) with 1X SYBR Safe (Invitrogen, California, USA).

### Site-directed mutagenesis in LCAT gene for plasmid vector construction

We used the GeneTailor Site-Directed Mutagenesis System kit (Invitrogen, California, USA) to obtain plasmids containing either the c.997G > A (p.V333 M) or c.1210A > G (p.M404 V) cDNA sequences, to be used as expression vectors for in-vitro cellular assays. Mutagenesis was performed using the pCMV6-XL4 circular vector carrying the cDNA sequence of LCAT (OriGene, Maryland, USA). Primers for the c.997G > A variant were: forward 5′-GCAGGACTCCCAGCACCTGGTATGGAAGTATAC-3′ and reverse 5′-ACCAGGTGCTGGGAGTCCTGCCAGGAGGTCACG-3′. Primers for the c.1210A > G variant were: forward 5′-CGGGATACAGCATCTCAACGTGGTCTTCAG-3′ and reverse 5′-GTTGAGATGCTGTATCCCGTGCAGGGGCAGC-3′. After transformation in *E. coli* DH5αTM-T1® (Thermo Fisher Scientific, California, USA), plasmid DNA was purified using AxyPrep Plasmid Miniprep (Axygen Biosciences, California, USA). Plasmid integrity was verified by enzymatic digestion with FastDigest® SmaI restriction enzyme (Fermentas, California, USA) and visualized on a 1% agarose gel. The presence of both c.997G > A and c.1210A > G variants were confirmed by direct sequencing using specific primers for this vector (VP1.5 and XL39, OriGene, Maryland, USA).

### Cell culture and expression vector transfection

Plasmids with wild-type, c.997G > A (p.V333 M) or c.1210A > G (p.M404 V) LCAT variants were transfected in HEK-293 T cells with the lipofectamine reagent (ThermoFisher Scientific, California, USA). Transfected HEK-293 T cells were cultured at 37 °C and 5% CO_2_ in D-MEM medium (Gibco, Gaithersburg, MD, USA) supplemented with 10% fetal bovine serum (Gibco) and 10% antibiotic mix (Penicilin/Streptomycin 10,000 U/mL) (Gibco).

### Western blot analysis

LCAT protein synthesis, expression and secretion was evaluated by Western blots in the plasma of the proband and relatives, as well as in cell lysates and supernatants of HEK-239 T cells. Total proteins were quantified with the BCA Protein Assay Kit (Pierce, Rockford, IL, USA). A total of 100 μg of protein/well was loaded in polyacrylamide gels for SDS-PAGE electrophoresis followed by electro transfer to nitrocellulose membranes and immunoblotting. For the detection of LCAT, rabbit polyclonal anti-LCAT antibody (Cayman, Ann Arbor, MI, USA) was used followed by incubation with goat anti-rabbit secondary HRP-conjugated antibody (Cayman). Luminol was used as a substrate for peroxidase and chemiluminescence was detected in a LAS-4000 image analyzer with a Fujifilm camera for exposed phase.

### Assessment of LCAT enzyme activity

LCAT activity was assessed by a fluorometric assay (Merck, Kenilworth, NJ, USA) in the plasma from the proband and relatives, as well as in supernatants of HEK-293 T cells. The estimation of the enzymatic activity was performed by plotting the fluorescence emission at 470/390 nm versus time. As reported from manufacturer, the absolute value of the magnitude of the negative slope for the 470/390 nm fluorescence measured across time is directly proportional to LCAT activity.

### Bioinformatic functional prediction and sequence conservation analysis

Multiple sequence alignment was performed using ClustalW to assess the conservation of amino acids in LCAT sequences across different species. Predictive bioinformatic online tools SIFT, PolyPhen2 and Condel were used to assess possible functional effects of p.V333 M and p.M404 V LCAT variants on LCAT function [9]. We also used MutPred to evaluate whether these exonic variants have effects on splicing [10].

### Molecular modeling and in-silico simulation analysis

Molecular models of wild-type (WT), p.V333 M and p.M404 V LCAT variants were constructed using MODELLER [11], as implemented in the Protein Modeling module of Discovery Studio v2.1 (Accelrys Inc., San Diego, CA). The human LCAT sequence retrieved from the Uniprot database (https://www.uniprot.org/uniprot/P04180) was used as reference and only residues 40–425 were considered for modeling purposes [12]. WT, p.V333 M and p.M404 V variants were modeled using crystal structures of human LCAT (PDB ids 4XWG and 5TXF) as templates [13, 14]. The LCAT WT, p.V333 M and p.M404 V systems were generated using VMD v1.93 [15], by inserting each protein into a 95 × 75 × 80 Å box consisting of classic TIP3P model for water molecules, and neutralized with Na^+^ or Cl^−^ ions [16]. Periodic boundary conditions were imposed in all three directions and the Particle Mesh Ewald method was used to account for full long-range electrostatic interactions within the selected boundary condition within a relative tolerance of 10^− 6^ [17]. The final systems were composed of nearly 54,000 atoms. Molecular dynamics simulations were carried out with the NAMD v2.10 simulation package [18], using the CHARMM36 force field parameters for proteins [19, 20]. The simulation was started with a brief energy minimization for 5000 steps, followed by 1 ns (ns) heating with protein backbone sequential release of alpha carbon restraints (force constants were gradually reduced from 10 kcal/mol Å^2^ to 0 kcal/mol Å^2^), 4 ns of equilibration and 50 ns production simulation for each protein was performed. The PME method was used for full long-range electrostatics within a relative tolerance of 1 × 10^− 6^. A 12 Å cut-off was used to compute non-bonded interactions with a smooth switching function applied at a distance of 10 Å. To impose the thermal exchange with an external thermostat, the isobaric-isothermal ensemble (NPT) with constant number of particles N, pressure P and temperature T was used. Constant temperature was maintained by coupling the system to a thermal bath whose temperature is maintained via Langevin dynamics with a friction coefficient of 1 ps^− 1^. Constant pressure was maintained using a Langevin piston at a nominal value of 1 atm [21]. The SHAKE algorithm, with a tolerance of 1 × 10^−8^Å, was applied to constrain the length of all covalent bonds involving hydrogen, thus allowing the use of a 2 femtoseconds integration time. Trajectory analyses and measurements were performed using VMD v1.9.3 [15]. By plotting Cα-root-mean-squared (RMSD-Cα) deviation and fluctuation (RMSF-Cα) along the MD simulation, we assessed the structural equilibration reached by the models.

### LCAT gene variants in unrelated participants of the growth and obesity Chilean cohort study (GOCS)

We evaluated the presence/frequency of the rare p.V333 M and p.M404 V variants, as well as the common p.L363 L (rs5923) variant in *n* = 741 children (Tanner stage = 1) from the pediatric population-based “Chilean Growth and Obesity Cohort Study” (GOCS). A detailed description of this cohort is available elsewhere [7]. The functional effect of the middle-low frequency p.L363 L variant (rs5923) was not functionally in vitro tested (as we did for p.V333 M and p.M404 V), given the synonymous nature of this polymorphism. Then, we planned to evaluate the possible association of rs5923 on plasma HDL-cholesterol levels in 848 pre-pubertal children aged 7.33 ± 0.87 y (50.1% female) from GOCS. The p.L363 L (rs5923) genotypes were retrieved from the Infinium® Multhi-Ethnic Global BeadChip (Illumina, San Diego, CA, USA) (MEGA-Illumina array). For quality-control purposes, genotyping data across the whole genome was loaded into GenomeStudio v2.0.3 (Illumina) and PLINK 1.9 to filter SNPs and samples based on call rate (exclude < 0.99), gender mismatch, relatedness, heterozygosity rate, ancestry outliers or extreme deviation from HWE [22]. Autosomal genetic markers contained in MEGA-Illumina were used to obtain Principal Components (PC) used to correct associations between rs5923 and plasma HDL-cholesterol for population stratification by ethnicity. To obtain such PC, high linkage disequilibrium (LD) regions were initially removed and were pruned using an independent pairwise approach [23] (window size of 50 Kb, a step size of 5 SNPs and a r^2^ cutoff threshold of 0.2). Principal Components (PCs) were computed in the obtained subset of 233,649 variants using the EIGENSTRAT method [24]. Association analysis between plasma HDL-cholesterol levels and rs5923 genotypes were carried through linear regression models incorporating gender, age, BMI and five PCs as covariates [25].

## Results

### LCAT sequence analysis in the family under study

Sanger sequencing identified three genetic variants in the exon 6 of the LCAT gene in the proband: the rare mutations p.V333 M (c.997G > A/ rs776035233) and p.M404 V (c.1210A > G/rs779114194), as well the middle-low frequency polymorphism p.L393 L (c.1188C > T; rs5923) (Additional file 1: Figure S1). In previous reports, p.V333 M was also referred as p.V309 M because it was not considered the initial 24-amino acid sequence that is removed during protein maturation [26]. Both parents and two siblings of the proband were found to be heterozygous carriers of only one of the two variants. Her mother and sister carry the p.V333 M LCAT mutation, while her father and brother carry the p.M404 V LCAT mutation. In the proband, we found a compound heterozygous genotype with p.V333 M and p.M404 V mutations (Fig. 1a). As mentioned in the “Subjects and Methods” section, none of her first-degree relatives of the proband suffered from any clinical symptoms of LCAT total and partial deficiency.

### Bioinformatic prediction of p.Val333Met and p.Met404Val LCAT variants

Multiple alignment of LCAT sequences from vertebrate species revealed that the p.V333 and p.M404 positions of exon 6 are highly conserved in diverse mammals and vertebrate species, in agreement with a possible relevance in LCAT function (Additional file 2: Figure S2). On the other hand, methionine at amino acid position 404 is also highly conserved and is located in a critical domain for correct folding, secretion, and maintenance of the active site of LCAT. Regarding predictive online tools for functional effect of missense variants, the p.Val333Met mutation was classified as “Probably damaging” by PolyPhen and “Deleterious” by Condel and SIFT. The p.Met404Val was classified as “Deleterious” by the SIFT program, but as “Neutral” or “Benign” by Condel and Polyphen respectively (Table 1). On the other hand, the MutPred software indicated that p.Met404Val mutation is possibly implicated in abnormal splicing.

**Table 1:** Bioinformatic predictive tools. Functional effect of LCAT mutations according to the bioinformatic predictive tools Condel, SIFT, PolyPhen, and MutPred

			Bioinformatic Predictive Tools			
Variant	SNP	Mutation type	Condel	SIFT	PolyPhen	MutPred
p.Val333Met	rs776035233	Missense	Deleterious	Deleterious	Probably Damaging	Abnormal Splicing
p.Met404Val	rs779114194	Missense	Neutral	Deleterious	Benign	Normal Splicing

### Molecular dynamics simulations

**Fig. 2:**
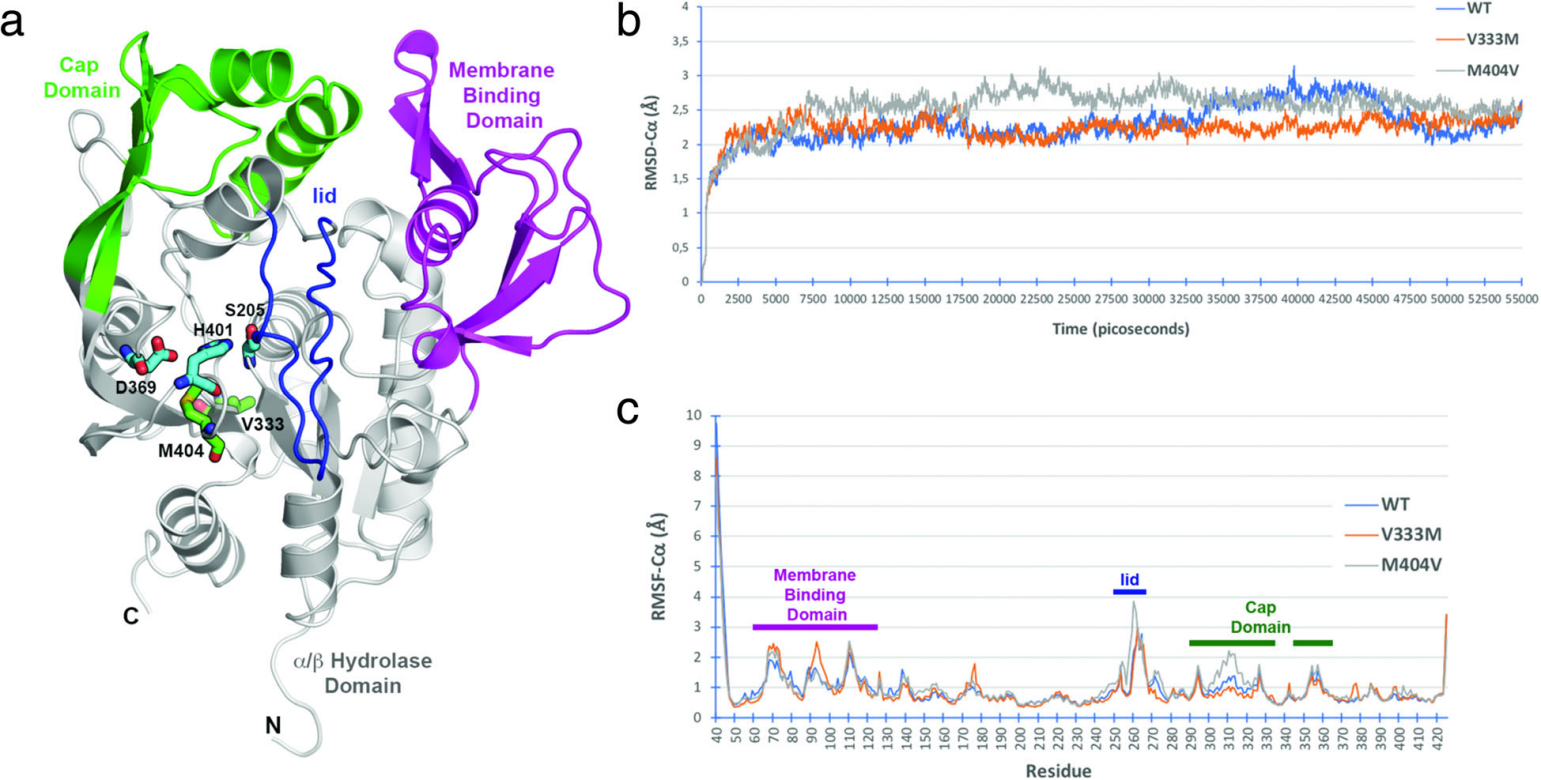
Molecular Dynamics Simulations of wild-type and variant LCAT. **a** Schematic representation of human LCAT (40–425) indicating important functional regions for activity and position of reported variants. **b** Root-mean square deviation of alpha carbons (RMSD-Cα) for WT and variant proteins during molecular dynamics. **c** Root-mean square fluctuation of alpha carbons (RMSF-Cα) for WT and variant proteins during molecular dynamics. Functional regions are depicted as bars with the color corresponding to panel “**a**”

To explore the structural and dynamic properties of WT and LCAT mutations, we carried out molecular *in-silico* dynamic simulations for LCAT protein in water. The p.M404 V mutation is located adjacent to the cholesterol binding site whereas the p.V333 M mutation occurs at the bottom of the α/β-hydrolase domain (Fig. 2a). Comparison of the RMSD (Root Mean Square Deviation) profiles indicated that the backbone atoms of WT as well as p.V333 M and p.M404 V mutations LCAT proteins stabilized after 5 ns, and only small deviations (< 1 Å) occurred (Fig. 2b). The areas with the highest conformational fluctuations were the N- and C-terminal ends, the cap domain and the membrane binding domain, as well as the lid domain that displays the greatest fluctuation if N- and C-terminal are not considered (Fig. 2c). The p.V333 M mutation presents higher flexibility in the 90–100 region within the membrane binding domain, while segment 300–325 display reduced mobility compared to WT and p.M404 V. On the other hand, variant p.M404 V shows increased flexibility in the membrane binding domain and lid regions. As hydrogen bonds and salt bridges play a role in conformational switching to generate active enzyme, we also evaluated the average distance of a number of hydrogen bonds and salt bridges. Distances between catalytic S205, D369 and H401 residues shows that both variants induce changes in the geometry of the active site and charge relay system with rigidification of the S205:H401 interaction in the case of p.M404 V variant (Additional file 3: Figure S3a and S3b). Changes in the substrate moving the away from the catalytic center as indicated by the loss of interaction between S273 and E173 (Additional file 3: Figure S3c). The high RMSD values for p.M404V lid domain correlate with the loss of interactions between M258 in the lid domain and residue G143 in the α/β-hydrolase domain (Additional file 3: Figure S3d).

### LCAT mutations p.V333 M and p.M404 V cause decreased enzyme activity in plasma and in vitro assays

**Fig. 3:**
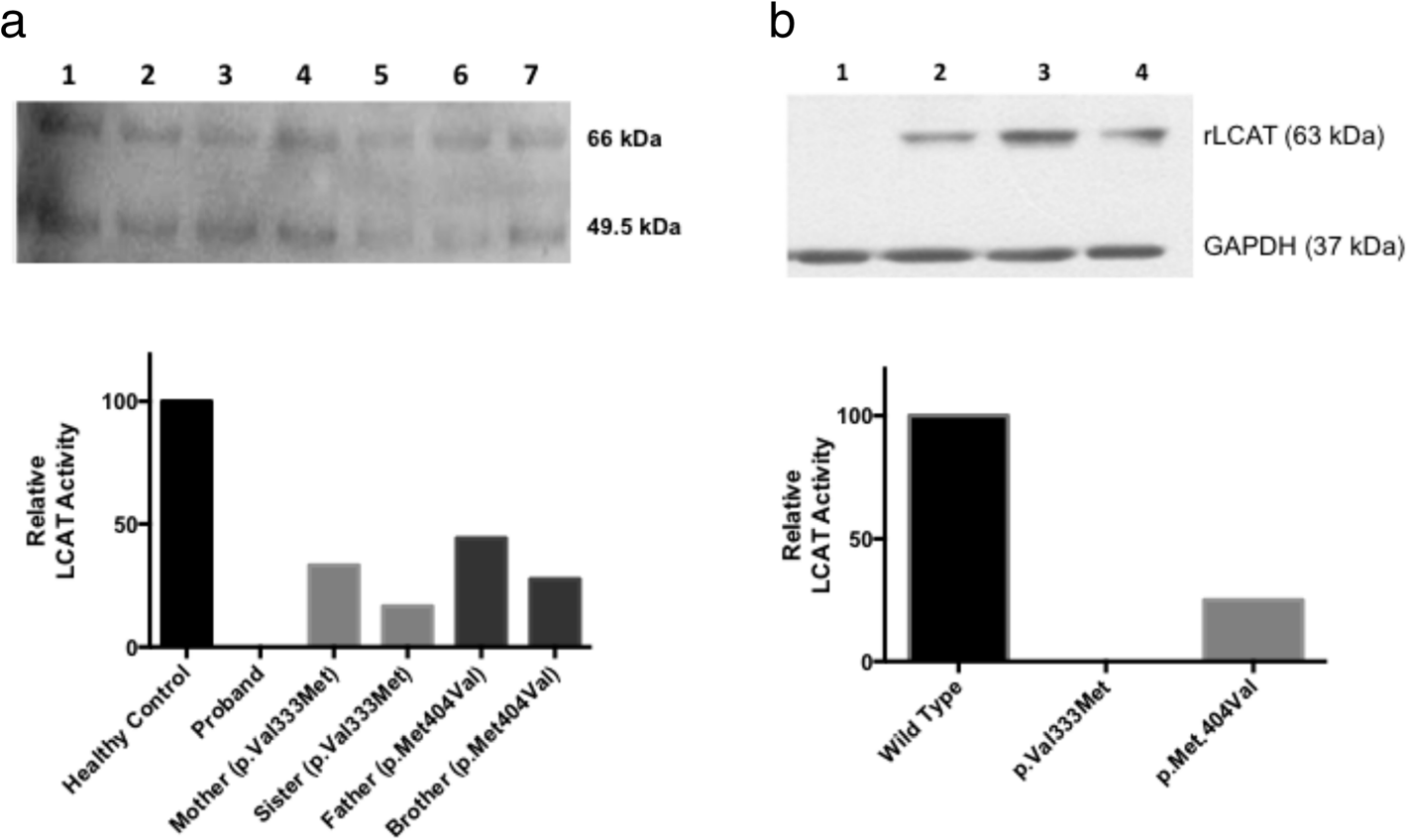
LCAT activity in human plasma and supernatant from transfected HEK-293 T cells. In **a** upper: lanes correspond to Father (1), Mother (2), Sister (3), Brother (4), Proband (5) and healthy controls (6 and 7). LCAT was detected as glycosylated (66 kDa) and non-glycosylated forms (49.5 kDa). In **a** bottom: relative LCAT activity is shown in plasma of the proband and her relatives. From left to right bars, they correspond to a healthy control, the proband (compound heterozygous p.V333 M/p.M404 V), Mother (p.V333 M carrier), Sister (p.V333 M carrier), Father (p.M404 V carrier) and Brother (p.M404 V carrier). Plasma from the proband did not show detectable LCAT activity. In **b** upper: lane 1 corresponds to mock, lane 2 corresponds to transfection with plasmids containing wild-type LCAT sequence, lane 3 corresponds to transfection with plasmids containing the p.V333 M LCAT mutation, and lane 4 corresponds to transfection with plasmids containing the p.M404 V LCAT mutation. The band at 63 kDa corresponds to recombinant LCAT (rLCAT) and 37 kDa to GADPH (control gene). In **b** bottom: relative LCAT enzyme activity is shown for supernatants from HEK-293 T cultured cells showing that both LCAT mutations showed decreased activity compared to LCAT Wild Type. In the graph, from left to right bars correspond to Wild Type LCAT, p.V333 M LCAT, and p.M404 V LCAT

After the identification of LCAT mutations p.V333 M and p.M404 V in the proband and in relatives, we determined whether these rare variants are related to LCAT amount and activity in plasma. First, we identified the positive presence of LCAT protein by Western blot in the plasma from the proband and her relatives (Fig. 3a). LCAT activity was virtually absent in the plasma from the proband, while plasma from her father and brother (carriers of the p.M404 V variant) showed reductions in LCAT activity by 56 and 72%, respectively compared to the control. On the other hand, plasma samples from her mother and sister (carriers of the p.V333 M variant), showed reductions of 67 and 83% reductions in plasma LCAT activity, respectively (Fig. 3a and Additional file 4: Figure S4).

Expression vectors pCMV6-XL4 carrying the WT LCAT, p.V333 M and p.M404 V were generated through site-directed mutagenesis and transfected into HEK-293 T cells. Expression, synthesis and secretion of LCAT were detected in cell lysates and supernatants by Western blot (Fig. 3b). No enzymatic activity was detected in the supernatants obtained from HEK-293 T cells transfected with the p.V333 M LCAT mutation. In contrast, enzyme activity of p.M404 V LCAT mutation decreased by 75% compared to cells transfected with the vector encoding WT LCAT (Fig. 3b and Additional file 5: Figure S5).

### Identification of LCAT gene variants in unrelated participants of the growth and obesity Chilean cohort study (GOCS)

We carried out a search of the p.V333 M and p.M404 V mutations through PCR-RFLP in those participants of the GOCS cohort with the lowest levels of HDL-cholesterol (< 40 mg/dL). In 57 subjects who met this criterion, none of the two mutations were detected, indicating that these mutations have a very low frequency in the Chilean population. On the other hand, the minor allele of the SNP rs5923 (p.L393 L) [27] was also found in the family under study in heterozygous state. This variant locates in the exon 6 of the LCAT gene with minor allele frequency of 4% in the Chilean population. As it is a synonymous variant, it was not possible to test its functional effect in the same way we did for p.V333 M and p.M404 V mutations through transfection of HEK-293 T cells. The presence of the minor allele correlates modestly but significantly with higher plasma HDL-cholesterol levels in our study group (*p* = 0.03) (Additional file 6: Fig. S6).

## Discussion

Herein, we identified the rare p.V333 M and p.M404 V mutations in the LCAT gene as a cause of LCAT deficiency in a Chilean patient with very low plasma HDL-cholesterol levels, corneal opacity, mild anaemia and multiple impaired lipid-related traits. The p.V333 M mutation was found to be deleterious for LCAT activity in reports from Italian [8], Polish [26] and Dutch [27] families, in which homozygous patients for this mutation significantly decreased plasma levels of HDL-cholesterol. In the Polish family, heterozygous members do not show clinical features of FLD, and LCAT activity was detectable, as it occurs in the Chilean family reported herein in which relatives show normal plasma HDL-cholesterol levels [26]. Molecular modeling suggest that the p.V333 M LCAT variant presents higher flexibility in the 90–100 amino acids region within the membrane binding domain, near to the critical Trp85 [14]. On the other hand, p.V333 M LCAT variant displays the higher distance between Ser273 and Glu173, and therefore substrate specificity is expected to be also affected [28, 29]. Moreover, valine 333 amino acid position of LCAT is highly conserved in mammals while its substitution to methionine in this position is predicted to have a deleterious effect by online prediction programs. All this observations and predictions are consistent with the phenotype found in the Chilean proband.

Our research is the first study that reports the functional effect of the rare c.1210A > G (p.Met404Val) mutation in the coding sequence of the LCAT gene. This amino acid position is also highly conserved in mammals and is located in a critical domain for correct folding, secretion, and maintenance of the active site of LCAT, being this region from the His401 located in the active site to the Asn408 glycosylation site (when not considering the signal peptide of 24 amino acids, methionine 404 is found in the codon 380) [4]. Although similar to p.V333 M in terms of its distance from the active site, the p.M404 V mutation decreases packing between the lid region and the α/β-hydrolase domain, suggesting that it may facilitates opening of the active site once LCAT approaches to HDL particles [30, 31].

The simultaneous presence of p.M404 V and p.V333 M in a compound heterozygous state is most likely to be the responsible of the undetectable LCAT activity observed in the Chilean proband assessed in this research. These variations in compound heterozygous state do not seem to affect plasma LCAT synthesis and secretion given the similar amount of enzyme detected in plasma or supernatant western blot. Carrying any of these variants separately did not show an effect on plasma HDL-cholesterol levels as found in proband’s first-degree relatives. However, it was very clear that the simultaneous presence of both mutations in the proband ablated plasma LCAT activity, with partial reductions in relatives carrying either p.M404 V or p.V333 M mutations. Additionally, site-directed mutagenesis, cell transfection in HEK-293 T cells, and functional analysis allowed the extensive in-vitro evaluation of the effect conveyed by p.V333 M and p.M404 V mutations in LCAT synthesis, secretion and activity. Although p.V333 M and M404 V LCAT mutations did not alter the synthesis or secretion of LCAT from cultured cells, they importantly decreased LCAT enzymatic activity. This effect was supported by predictive online analysis and molecular dynamic simulations. Therefore, p.V333 M and p.M404 V mutations are causative of the metabolic disorders associated with severe LCAT deficiency in the proband. Reduced activity of p.V333 M LCAT is consistent with in vitro tests carried out by Calabresi et al in transfected COS-1 cells. Taken together, this evidence indicate that such sequence variation may lead to the clinical manifestations of LCAT deficiency [32].

Another variant found in the Chilean proband is the presence of the minor allele of the p.L393 L polymorphism of LCAT gene. This is a middle-low frequency variant found in 4% of Chileans. As p.L393 L does not change amino acid sequence it was not possible to assess its in vitro effect in the same way as we did for p.V333 M and p.M404 V. It was previously reported a lack of association was found between p.L393 L variant with in LCAT activity or HDL-cholesterol levels [33, 34]. Surprisingly, we found a modest although significant nominal association between p.L393 L and plasma HDL-cholesterol levels in GOCS participant. However, it is very unlikely that this variant has an important effect in the hypoalphalipoproteinemia found in the proband of the family under study based on its synonymous nature, its relatively high frequency in the population, its reported lack of effect on LCAT activity, and the low magnitude of its putative association with plasma HDL-cholesterol levels.

## Conclusion

Two rare mutations of LCAT gene, p.V333 M and p.M404 V, were identified in a Chilean patient with hypoalphalipoproteinemia, corneal opacity, mild anemia and other impaired lipid-related clinical traits. Based on intensive biochemical, genetic, bioinformatic and in vitro LCAT analysis, we conclude that these mutations abolish cholesterol esterification activity catalyzed by LCAT and are responsible of the clinical features of Familial LCAT deficiency found in this patient. The clinical significance for each variant was deposited in ClinVar under the access numbers SCV000899249 for p.M404V and SCV000899250 for p.V333M

## Additional files

Additional file 1: **Figure S1.** LCAT genetic variants found in the proband. Sanger sequences are shown for L393 L (upper panel), V333 M (middle panel) and M404 V (bottom panel). (JPG 77 kb)

Additional file 2: **Figure S2.** Multiple alignment of LCAT across species. Sequence of the exon six of LCAT gene from different species are shown. Conserved amino acid residues between species in position 333 and 404 are highlighting in red boxes. (JPG 1002 kb)

Additional file 3: **Figure S3.** Distances between selected amino acid residues during molecular dynamics simulations of wild-type and mutant LCAT. **(**a) Distance between catalytic residues Ser205 (side chain O) and His401 (epsilon N). (b) Distance between catalytic residues Asp369 (side chain O) and His401 (delta N). (c) Distance between charge relay system residue Glu173 (side chain delta C) and Ser147 (side chain O). (d) Distance between residues Met258 (main chain O) in the lid region and Gly143 (main chain N). (TIF 827 kb)

Additional file 4: **Figure S4.** Plasma LCAT enzyme activity of the proband and first-degree relatives. Plasma LCAT enzyme measurements were performed in a healthy control, the proband and her first-degree relatives. For each sample, ratio between emission intensity 470/390 nm versus reaction time was represented. LCAT activity is directly proportional to the absolute value of the slope. (JPG 354 kb)

Additional file 5: **Figure S5.** LCAT enzyme activity in supernatants of transfected cells HEK-293 T cells. Enzyme activity measurements in the supernatant of transfected cells with wild-type LCAT sequence, and mutations p.V333 M and p.M404 V were performed. For each sample, ratio between emission intensity 470/390 nm versus reaction time was graphed. LCAT activity is directly proportional to the absolute value of the slope. (JPG 289 kb)

Additional file 6: **Figure S6.** Association of p.L363 L LCAT variant with HDL-cholesterol levels in GOCS. The graph shows the relation between p.L363 L genotypes and plasma HDL-cholesterol levels in the GOCS cohort. Homozygous for the major allele is indicated as CC, heterozygous is CT, and homozygous for the minor allele is the TT genotype. (JPG 28 kb)

Additional file 7: **Table S1.** Plasma cholesterol-ester vs cholesterol-free ratios in the proband. Table shows cholesterol-ester (CE) vs cholesterol-free (CF) ratios in VLDL, LDL and HDL fractions determined by FPLC in the proband compared to normal reference values of controls. These results indicate very low levels of cholesterol-ester in the proband, which is compatible with a sharp reduction in LCAT activity. (PDF 11 kb)

## Abbreviations

FLD: Familial LCAT deficiency
FPLC: Fast-Protein Liquid Cromatography
GOCS: Growth and Obesity Cohort Study
HDL: High-density lipoproteins
LCAT: Lecithin-cholesterol acyltransferase
LDL: low-density lipoproteins
RFLP: Restriction Fragment Length Polymorphism
RMSD: Root Mean Square Deviation
VLDL: Very low-density lipoprotein
WT: Wild-type
