# Supplementary figures and images for: Identification and functional analysis of missense mutations in the lecithin cholesterol acyltransferase gene in a Chilean patient with hypoalphalipoproteinemia
Source: Lipids Health Dis. 2019 Jun 5;18:132. doi: 10.1186/s12944-019-1045-0 (PMC6549291; doi:10.1186/s12944-019-1045-0)

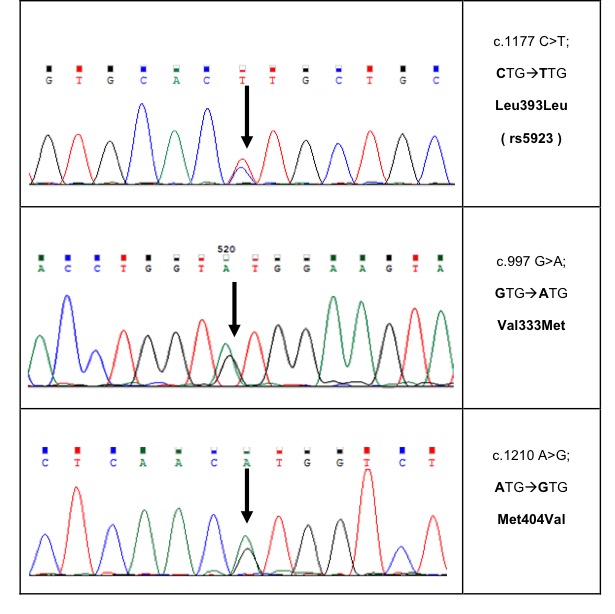

Supplement: Supplementary file 1 — Figure S1. LCAT genetic variants found in the proband. Sanger sequences are shown for L393 L (upper panel), V333 M (middle panel) and M404 V (bottom panel). (JPG 77 kb) [file 12944_2019_1045_MOESM1_ESM.jpg]

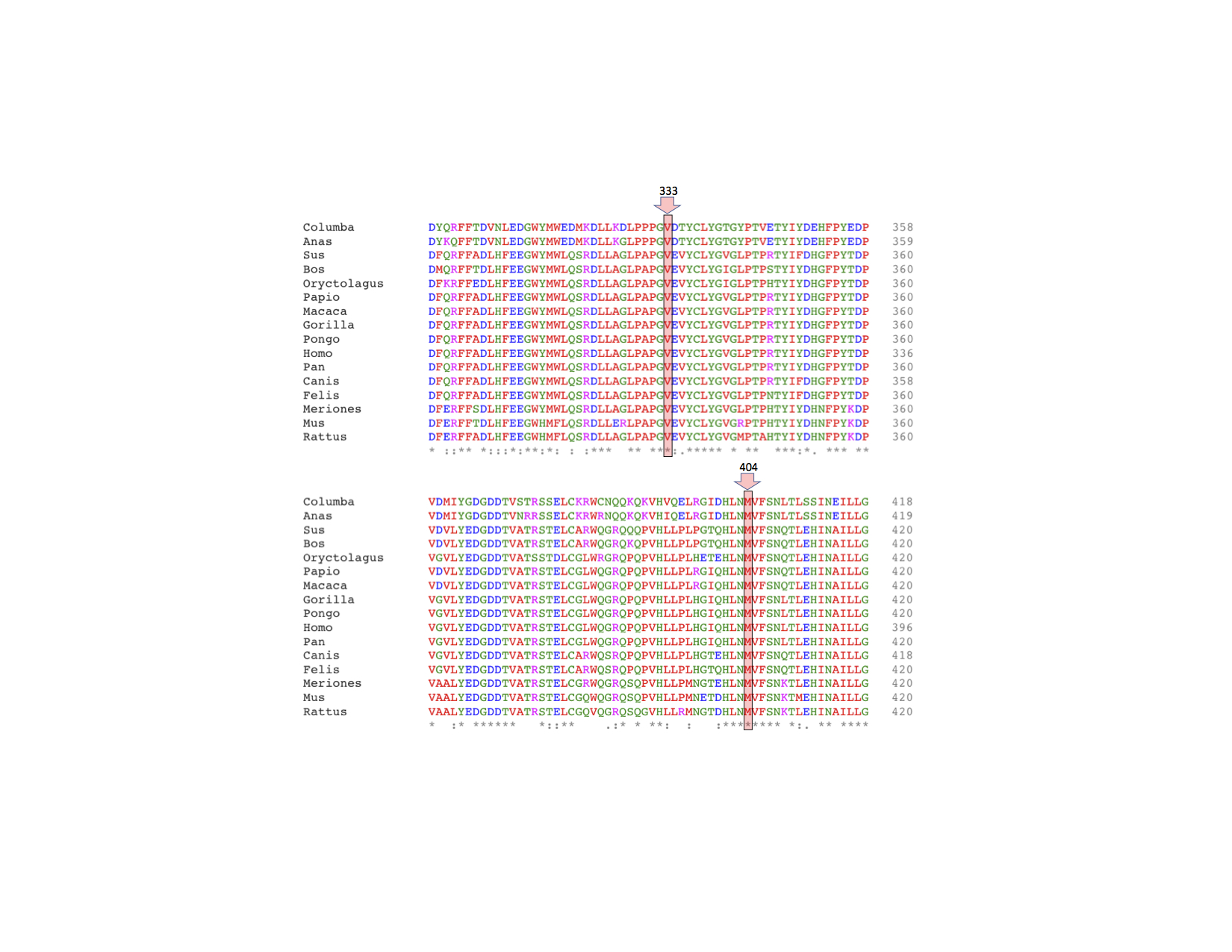

Supplement: Supplementary file 2 — Figure S2. Multiple alignment of LCAT across species. Sequence of the exon six of LCAT gene from different species are shown. Conserved amino acid residues between species in position 333 and 404 are highlighting in red boxes. (JPG 1002 kb) [file 12944_2019_1045_MOESM2_ESM.jpg]

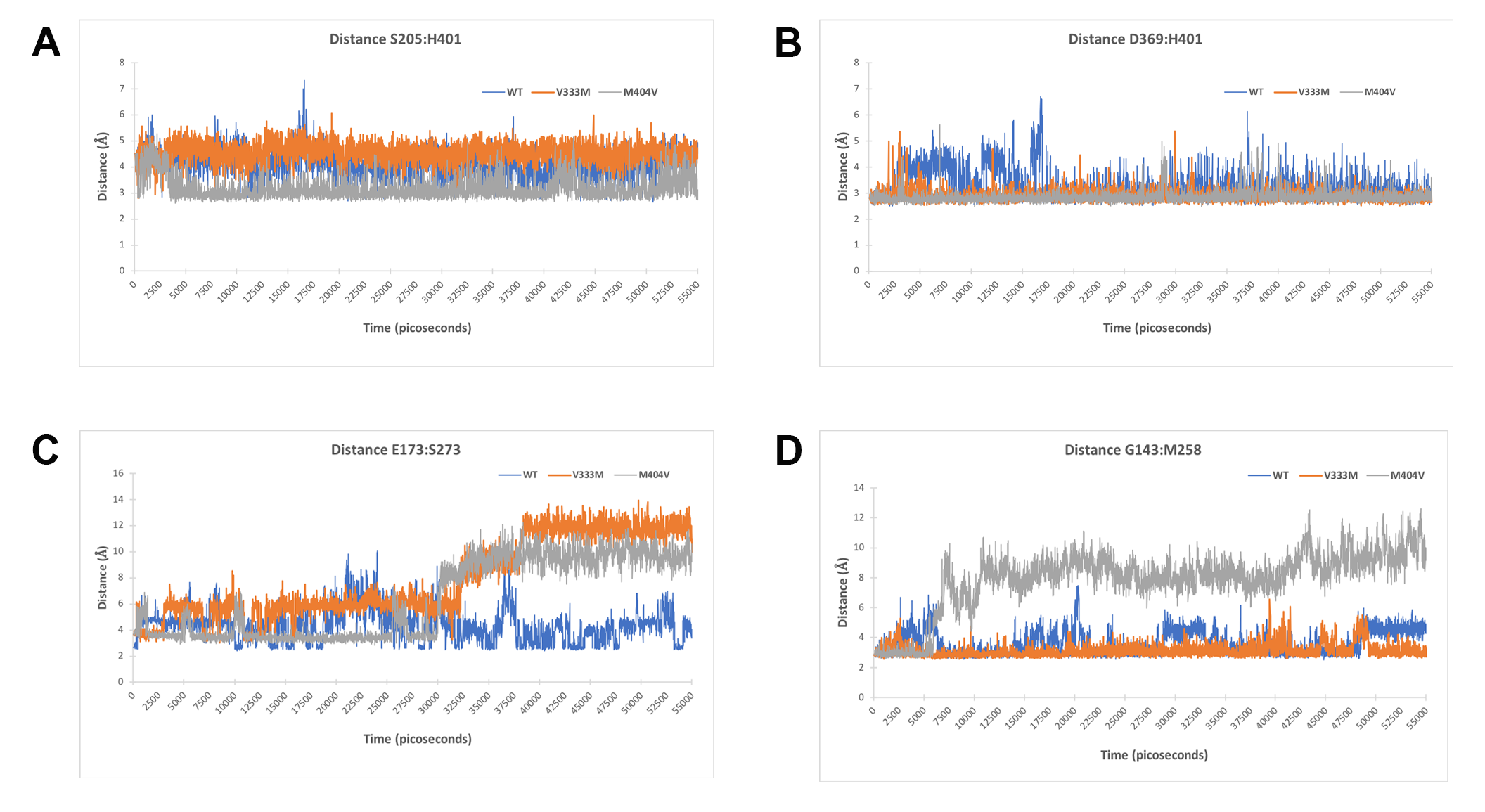

Supplement: Supplementary file 3 — Figure S3. Distances between selected amino acid residues during molecular dynamics simulations of wild-type and mutant LCAT. (a) Distance between catalytic residues Ser205 (side chain O) and His401 (epsilon N). (b) Distance between catalytic residues Asp369 (side chain O) and His401 (delta N). (c) Distance between charge relay system residue Glu173 (side chain delta C) and Ser147 (side chain O). (d) Distance between residues Met258 (main chain O) in the lid region and Gly143 (main chain N). (TIF 827 kb) [file 12944_2019_1045_MOESM3_ESM.tif]

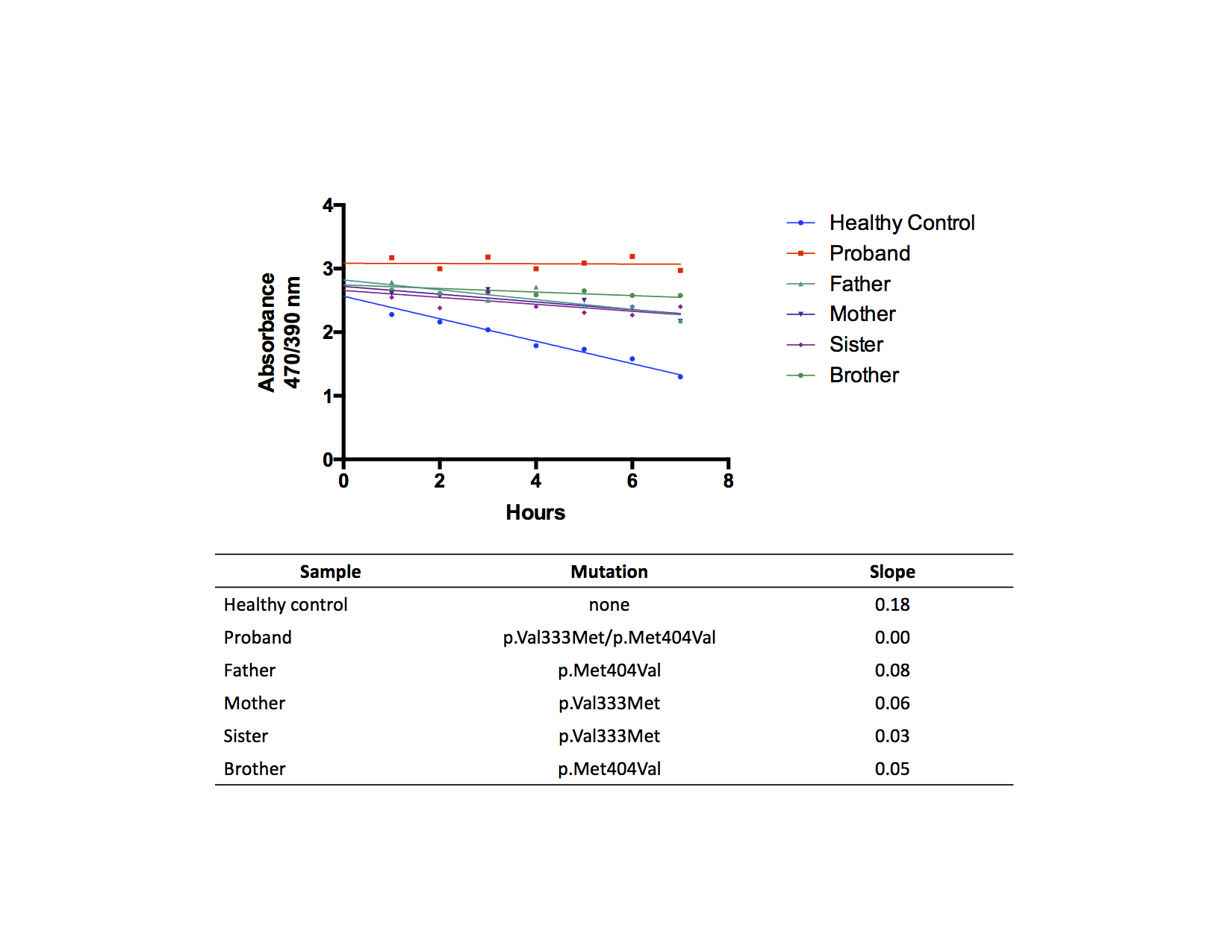

Supplement: Supplementary file 4 — Figure S4. Plasma LCAT enzyme activity of the proband and first-degree relatives. Plasma LCAT enzyme measurements were performed in a healthy control, the proband and her first-degree relatives. For each sample, ratio between emission intensity 470/390 nm versus reaction time was represented. LCAT activity is directly proportional to the absolute value of the slope. (JPG 354 kb) [file 12944_2019_1045_MOESM4_ESM.jpg]

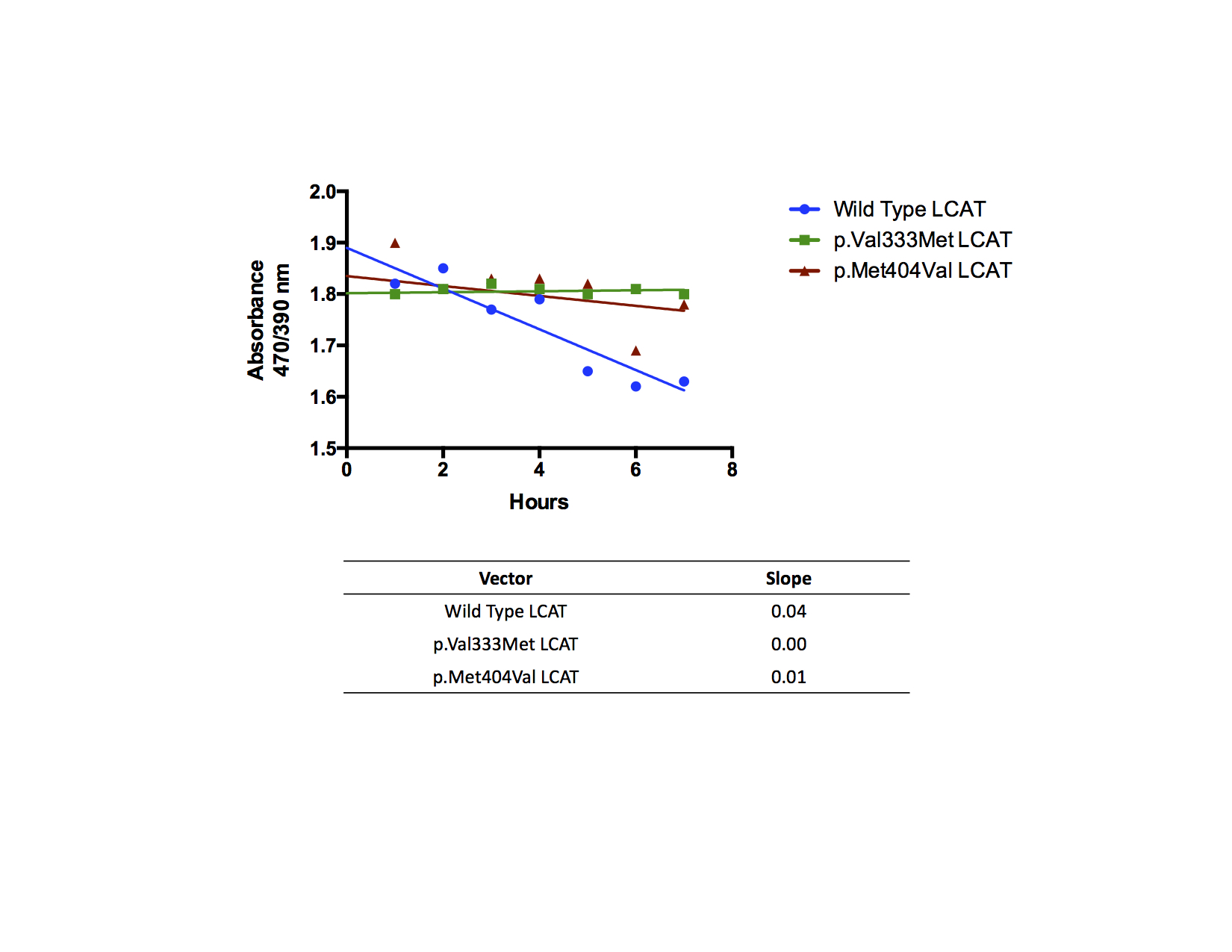

Supplement: Supplementary file 5 — Figure S5. LCAT enzyme activity in supernatants of transfected cells HEK-293 T cells. Enzyme activity measurements in the supernatant of transfected cells with wild-type LCAT sequence, and mutations p.V333 M and p.M404 V were performed. For each sample, ratio between emission intensity 470/390 nm versus reaction time was graphed. LCAT activity is directly proportional to the absolute value of the slope. (JPG 289 kb) [file 12944_2019_1045_MOESM5_ESM.jpg]

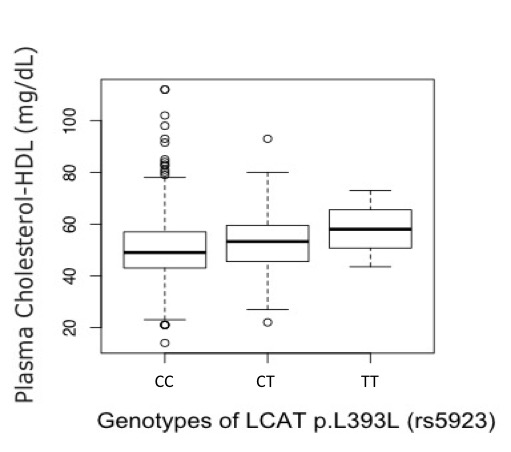

Supplement: Supplementary file 6 — Figure S6. Association of p.L363 L LCAT variant with HDL-cholesterol levels in GOCS. The graph shows the relation between p.L363 L genotypes and plasma HDL-cholesterol levels in the GOCS cohort. Homozygous for the major allele is indicated as CC, heterozygous is CT, and homozygous for the minor allele is the TT genotype. (JPG 28 kb) [file 12944_2019_1045_MOESM6_ESM.jpg]
