# Supplementary material for: Identification and functional analysis of missense mutations in the lecithin cholesterol acyltransferase gene in a Chilean patient with hypoalphalipoproteinemia
Source: Lipids Health Dis. 2019 Jun 5;18:132. doi: 10.1186/s12944-019-1045-0 (PMC6549291; doi:10.1186/s12944-019-1045-0)
Supplement: Supplementary file 7 — Table S1. Plasma cholesterol-ester vs cholesterol-free ratios in the proband. Table shows cholesterol-ester (CE) vs cholesterol-free (CF) ratios in VLDL, LDL and HDL fractions determined by FPLC in the proband compared to normal reference values of controls. These results indicate very low levels of cholesterol-ester in the proband, which is compatible with a sharp reduction in LCAT activity. (PDF 11 kb) [file 12944_2019_1045_MOESM7_ESM.pdf]

	Proband	Normal range
CE/CF in VLDL	0.1	1.0 – 3.0
CE/CF in LDL	0.3	3.5 – 5.7
CE/CF in HDL	0.2	3.0 – 4.0
